# Factors that influence the use of community assets by people with physical disabilities: results of participatory mapping in Envigado, Colombia

**DOI:** 10.1186/s12889-020-8285-9

**Published:** 2020-02-04

**Authors:** María Luisa Toro-Hernandez, Laura Villa-Torres, Mónica Alejandra Mondragón-Barrera, Wendy Camelo-Castillo

**Affiliations:** 1 School of Physical Therapy, CES University, Medellin, Colombia; 2 Department of Social Medicine, UNC School of Medicine, University of North Carolina at Chapel Hill, Chapel Hill, USA; 3 Department of Pharmaceutical Health Services Research, School of Pharmacy, University of Maryland Baltimore, Baltimore, USA

**Keywords:** Disability, Contextual factors, Community asset mapping, Social participation

## Abstract

**Background:**

Disability is an evolving concept that results from the complex interaction between a person with an impairment and the context in which he/she lives. There is limited understanding on the types, access and use of community assets valuable for people with disabilities, and the role of contextual factors in Colombia. Our goal with this work was to identify the factors at the levels of the socio-ecological framework, and their interaction, that influence the use of community assets among people with physical disabilities and community stakeholders in Envigado, Colombia.

**Methods:**

Using participatory mapping, a community based participatory approach, we carried out an assessment of community assets identified by people with disabilities and rehabilitation professionals. In-depth interviews (*n* = 32) informed the design of two participatory mapping activities, one among people with disabilities (*n* = 5) and a second with rehabilitation professionals (*n* = 4). Results were presented in a community forum to receive feedback on the findings.

**Results:**

Main findings indicate a chain of contextual factors that limit access and use of assets stemming from the personal (e.g. financial resources, inaccessible housing), interpersonal level (e.g. lack of a personal assistance or aid), and community levels (e.g. lack of accessible public transportation and inaccessible buildings). In most cases these barriers are heightened by system level barriers (e.g. lack of effective enforcement of the legal framework).

**Conclusions:**

Identifying these contextual factors, and their interactions, calls for stronger enforcement of the existing legal framework through articulated work between different stakeholders, so that people with disabilities can enjoy community assets.

## Introduction

People with disabilities represent 1 in 7 adults, or 15% of the world’s population.^1^[1] Since the enactment of the United Nations Convention of the Rights of Persons with Disabilities (CRPD) in 2006, disability has become a global human rights and development priority [1–3]. Despite this, many people with disabilities still face significant attitudinal, physical, communication, and information barriers that hinder their full participation in their communities as well as the exercise of their human rights [1]. Over the last two decades, Colombia has made significant rights-based policy advances to improve participation and reduce inequalities among individuals with disability, including the ratification of the CRPD [4]. Since the passage of the first law to promote the rights of people with disabilities in 1997, national policies have been enacted to promote accessibility of the public built environment and transportation, mandate inclusive education, and support affirmative action to provide equal opportunities for employment [5–8]. In spite of these advances, in 2016 the first shadow reports to the United Nations on the CPRD implementation acknowledged that Colombians with disability face significant discrimination, low access to education and low employment rates, with larger inequalities experienced by those living in non-urban areas [9, 10]. The limited available literature suggests that there is a gap between the implementation of disability-related policies and the meaningful participation of people with disabilities in their societies [2].

Given than the disability experience is multifactorial, the interaction between individual characteristics, behaviors and the context may result in limited access and use of community assets [11]. Community assets are defined as anything that can be used to improve the quality of life in the community including places, services, businesses, and people [12]. Through access and use of community assets, participation of people with disabilities is increased. Studies in developed countries have reported that contextual factors often intersect and have cumulative influences on the ability of people with disabilities to fully participate and use community assets [13]. For instance in New Zealand, the interaction between factors at the personal, interpersonal, community, and policy level such as motivation, accompanying individuals, negative attitudes from service providers, and governmental funding limited access to sports and recreational community assets [14]. In the United States, barriers within the built and natural environment, finances, assistive technology, transportation, information and technology access, social support and attitudes, systems and policies, affected participation [13].

Characteristics of the social and physical context can either have a positive or negative effect on the well-being and participation of people with disabilities. Under the socioecological framework it is recognized that barriers to participation cannot be only explained by individual characteristics. The broader social context that ranges from the micro-level (including family, neighborhood and extended social networks) to the macro-level (stigma, discrimination, system policies) can hinder or enhance individual agency that leads to participation. Efforts to increase participation of people with disability have largely focused on the individual, overlooking the role of interpersonal, community and system level factors, as well as their interactions [2, 15]. Alongside with the growing recognition of the role of socio-ecological factors and the importance of developing interventions that target multiple levels, there is a need to understand how social and physical factors interact at each level to limit participation among people with disability, within specific contexts [2, 16]. In Latin America there is limited evidence and documentation on how community assets are accessed and used. Using a socio-ecological framework and a community based participatory approach our goal with this work is to identify factors at the individual, interpersonal, community and system levels that limit access and use of community assets among people with disability in Colombia.

## Methods

### Setting

This work was developed in partnership with the community organization Alfime during 2017. Alfime offers educational programs to people with disabilities and their families, as well as rehabilitation health services (physical therapy, physical activity, psychology, legal advice, and independent living programs). Most of the programs are subsidized through public funds from the city of Envigado, located in the metropolitan area of Medellin, Colombia. Medellin is the second largest city in the country. The northern border of Envigado is Medellin and its downtown is located ten kilometers away from Envigado’s downtown. Alfime is one of the main resources available for people with disabilities in Envigado. On average, Alfime provides services to 250 people with disabilities every year; the city disability registry reported 1975 people with disabilities in Envigado in 2015 [17]. Nonetheless, while not the majority, Alfime also provides services to people with disabilities from surrounding cities.

### Sample and recruitment

The results presented in this paper are part of a larger study that aimed to understand the barriers people with disabilities face to access rehabilitation services and other social services in Envigado. Our study was conducted in three stages: individual qualitative interviews, a participatory mapping activity, and the socialization of findings through a community forum. In the first stage of this study we identified a purposive sample (*n* = 32) of people with disabilities, caregivers, rehabilitation professionals, and community leaders to characterize the factors and levels of influence that limit access and use of community assets by individuals with physical disability [18]. People with disabilities were defined as having a permanent physical or mobility impairment affecting their body, upper or lower limbs, dexterity or coordination [19]. We focused on people with physical impairments as they represent the largest proportion of people with disabilities in the country [19]. People with disabilities in our study had to be affiliated with Alfime, ages 18–44 and residents of Envigado, Antioquia. Caregivers and rehabilitation professionals had to provide care for people with disabilities or be involved in services and programs aimed at improving functioning among this population. Rehabilitation professionals were chosen as key informants for this stage as their knowledge and awareness of the community assets may foster to match individuals with disabilities to opportunities in their community [20]. Based on the themes identified through the qualitative interviews (results and procedures published elsewhere) [18] we further explored the role of the context and its interaction with factors that limit the use of community assets, during the participatory mapping activity. For this second stage, we only included people with disabilities (*n* = 5) and rehabilitation professionals (*n* = 4). The Institutional Review Board of the CES University reviewed and approved this study. Informed consent was obtained from all participants in the study.

### Procedures

For the first stage of this work we conducted in-person semi-structured interviews in Spanish. The interview guide explored the areas of independence and autonomy, access to resources, and citizenship. Interviews took place at Alfime or a location preferred by the participant.

For the second stage of the study we used participatory mapping as a method that allows the identification of community assets, and potential facilitators and barriers for their use and access [21]. Participatory mapping is a visual and didactic tool that allows the dialog among community members and stakeholders to establish a vision of their own community [22–24]. This method allows to go beyond descriptions (e.g. in-depth interviews) and to graphically build with collective knowledge the complexity of their community [25]. It is used as a tool to understand and articulate factors in communities that seem isolated [26, 27]. This may be used as a baseline to plan for interventions and measure progress [28].

Based on the results from the in-depth qualitative interviews (published elsewhere), we identified the central themes for the participatory mapping activity [18]. People with disabilities (*n* = 5) and rehabilitation professionals (*n* = 4) were invited to participate in two sessions. During the first session the instructions for the mapping activity were given. Participants needed to identify community assets, defined as places they deemed valuable for their lives and that they regularly visited. Instructions included showing participants how to record the entries in an activity sheet, where they were asked to record all locations they went to during the week following the session, including name of places visited, addresses, routes and transportation means used to get there, assistance required, and a description of accessibility at the final destination.

A week later, a second group session was conducted where each participant shared their entries. Using a large-scale map of the city of Envigado, participants were asked to place the assets on the map and describe routes and transportation means used to get there, assistance required, and a description of the accessibility at the final destination. During the discussion participants elaborated on their daily life during the week and reflected on past experiences that may had not been recorded but that were of value. The discussions were held separately for people with disabilities and rehabilitation professionals. Discussions were digitally recorded with participant’s permission. The group sessions were facilitated in Spanish by two of the research team members [MLTH and MAM]. These discussions took place at a private room in Alfime. They lasted 90 min for rehabilitation professionals and 100 min for people with disabilities. Field notes were completed during and following each group discussion.

In the third stage of this work, findings from the in-depth interviews and participatory mapping were disseminated to a group of stakeholders in Envigado through a community forum. Dissemination of findings was done in partnership with people with disabilities who participated in the study. Local government officials, rehabilitation professionals, people with disabilities and their families, and the academia were invited to the forum. More than 40 people participated in the forum and provided feedback.

### Data analysis

**Fig. 1:**
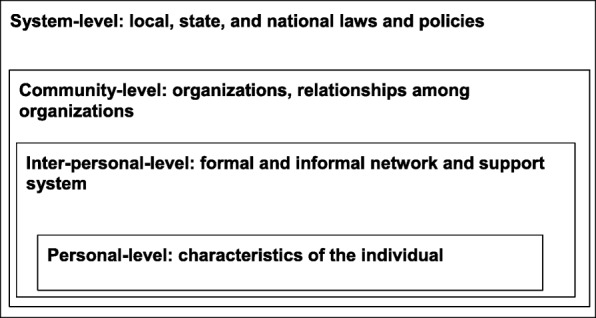
Four levels of the socio-ecological framework used by Mulligan et al. [14] which adapted it from the social ecology of health promotion interventions from Mcleroy et al. [29]

All group sessions were transcribed verbatim and data was managed and analyzed using Dedoose Version 8.0.35, web application (2018). We used thematic content analysis to analyze the data from in-depth interviews and participatory mapping [25]. Techniques used in the analysis included analytical summaries, open coding, identification of thematic codes, and codebook development. Each group discussion was coded by two members of the team; disagreements were discussed and resolved through involvement of a third team member. Through thematic content analysis we identified community assets, major challenges regarding access, and use of assets. Using the socio-ecological framework we identified barriers and their interactions, and placed them at the personal, interpersonal, community and system level that limit access and use of community assets (Fig. 1). The socioecological framework has been used to explore the interactions of factors at the personal, interpersonal, organizational/community, and socio-political levels, and to characterize outcomes related to inclusion of people with disabilities [14, 30, 31]. Through this framework the dynamic and interdependent interactions of individuals, their immediate settings, and the formal and informal larger social and physical contexts (assets) can be explored [32–34]. Data saturation was evaluated using an iterative analytical process that included reviewing field notes, reading and coding the data, and developing analytical matrices. Our saturation assessment along-side with data triangulation across participants gives us confidence that the key themes were saturated.

## Results

A total of forty-one participants took part in the larger study. Thirty-two in the in-depth interviews and nine in the participatory mapping exercise, plus forty people attended the community forum. The demographics characteristics of the in-depth interviews are described in detail elsewhere [18]. To protect the confidentiality of the participants we only provide the overall demographic characteristics of people with disabilities and rehabilitation professionals: all but one participant lived in Envigado, seven were women, and five were wheelchair users.

**Fig. 2:**
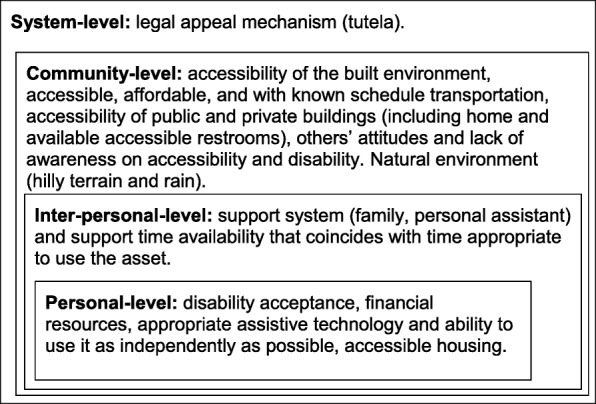
Factors at the personal, and interpersonal, community, and system levels that influence access and use community assets by people with physical disability in Envigado, Colombia [Figure developed by the authors]

Overall, participants in the participatory mapping exercise identified a series of factors at the personal, interpersonal, community, and system levels that limited access and use of assets in the community (Fig. 2). Only rehabilitation professionals mentioned that the lack of individual disability awareness and self-acceptance hinders the enjoyment and use of community assets. This personal factor was not mentioned by the group of people with disabilities.

Both people with disabilities and rehabilitation professionals indicated that the mapping exercise made them realize that they visited several places in their day-to-day life and acknowledged the vast number of physical barriers to access an asset in their community. Assets identified in the community included places related to health (providers facilities), sports and recreation (theaters, shopping malls, stadiums, gyms, bars and restaurants, public parks), public services (city hall, notary services, banks), private buildings (one’s home and family/friends home), religious worship places, education, and grocery stores. Only rehabilitation providers mentioned banks and notaries and one person with disability mentioned the airport. Most of the assets were in Envigado (suburban) except specialized health care services that were in Medellin (urban).

Participants acknowledged that there are accessibility efforts at the community level; however, people’s attitudes and behaviors hinder the use of assets by people with disabilities. There may be accessible public spaces, but the inappropriate use of the space by others in the community makes them inaccessible. An example is when vehicles are parked blocking sidewalks curb cuts. Testimonies in both groups depict community accessibility measures that fail to facilitate the enjoyment of assets:

*“Now that we are talking about San Rafael hospital, there is a ramp but it is too steep, making it very difficult to go up” [Male, Person with disability].*

*“To enter the theater there are a lot of stairs, there is a stair lift but it only fits one person...if you go with a group of people that needs the lift … how long do you have to wait?..it also needs to be operated by someone from the theater, resulting in prolonged waiting times to access” [Female, rehabilitation professional]*

Interactions between the factors exacerbate the barrier(s) experienced at any given level, resulting in exclusion. In situations when people apparently have access to assets in their communities, the existing barriers result in not using them at all. For example, one of the rehabilitation professionals that also lives with a disability quit school because classes were at night and it was dangerous for him going back home on his wheelchair on the road: *“Last semester was very hard, it was at night (classes) and that is why I quit school...going back home rolling...more than one (car) will honk on me … and raining” [Male, Rehabilitation professional]*. In some cases, people with disabilities simply do not leave their homes as sorting out the barriers is too complex. This is reflected in the experiences of participants in both groups:

*“I’m a soccer fan, here is difficult to enter the stadium. They let me in; but, I have to be at the lawn by myself … .So* ***I stopped going*** *and now I watch the games by myself at home. All my family goes to the stadium and I have to stay behind at home” [Female, Person with disability]*

### Interaction of factors at different levels to access and use community assets

Enjoying and using assets in their communities (i.e. participating) is determined by the ability to simultaneously manage factors at different levels. Strategies to overcome situations that result from the interaction of factors at different levels from both groups were abundant. We provide and discuss examples for each levels, as follows.

**Personal-Interpersonal**: the lack of an accessible home and an appropriate wheelchair increases the need for assistance and financial resources as it requires extra costs (e.g. paying someone for assistance to leave the home or to get to the asset of interest).

*“*“*I prefer the church that is closer to my home...when it is not raining and my two sons are at home...I’m happy that they take my power wheelchair and I can go by myself to church...My daughter can’t take it down, it is too heavy...” [Female, Person with disability]*

The accessibility of the community access does not suffice, if the person with a disability needs support from another individual, the time when the asset needs to be used must coincide when the time that the support person is available:

*“I stopped going to a micro-enterprise course because the person that goes with me can’t always go...if she can’t go with me, I have to pay for transport...expenses are higher than income...people believe that it is just a matter of enrolling in an activity...but you have to do more things than that … ” [Female, Person with disability]*

A similar case was described by a professional who did a home visit that week:

*“...That person’s home has the worse accessibility, a 5th floor, no elevator and no ramp...two relatives have to leave work early once a week to carry him up and down the stairs in his wheelchair so he goes out” [Female, Rehabilitation professional].*

Interactions between personal factors such as living situation and interpersonal factors such as an assistant’s support result in hindered participation.

**Personal-Community:** Lack of accessible and reliable public transportation may increase the need for financial resources (e.g. to pay a taxi), to have extra time (e.g. not knowing when the accessible bus comes through the bus stop) or having to roll long distances to get to the asset of interest.

*“There are some public buses with accessibility [a lift for wheelchairs], not all of the buses have and we do not know with what frequency they run. This forces me to pay for taxi, I can’t be late for an appointment and I can’t go rolling” [Female, Rehabilitation professional]*

Lack of physical accessibility in routes to get to an asset requires advanced wheelchair mobility skills to navigate obstacles. When reflecting on the effect that lack of physical accessibility has on the participation of wheelchair users, only rehabilitation professionals mentioned that to be able to access assets, wheelchair users must learn advanced wheelchair mobility skills. As described by a male rehabilitation professional:

*“I usually roll [the manual wheelchair] on the street....accessible sidewalks...very few...even the new ones that we evaluated the other day are too high and do not have a curb cut”. [Male, Rehabilitation professional]*

In this specific case the participant is able to overcome the physical obstacles because he has advanced wheelchair mobility skills. In the discussion about community assets related to leisure and culture, one provider stressed:

*“We have the house-museum...to get there...people definitely need to learn how to maneuver their wheelchairs [the entrance is through a gravel parking lot]” [Female, Rehabilitation professional]*

Lack of accessible facilities, including restrooms, require the person to plan or overcome extra logistics (e.g. identifying an accessible restroom that may be at a different floor and taking longer routes to avoid obstacles). Lack of accessible and continuous pathways to go from one place to another may result in the person having to take more risks (e.g. roll the wheelchair on the street with the cars and motorcycles, having to be lifted by others up/down curbs or stairs).

*“The Nueva EPS [health center] has a mini ramp, but the doctors’ offices are in the second floor. That is why they have to see you downstairs … ” [Female, Rehabilitation professional]*

Participants acknowledged that there is progress and that some public spaces in Envigado have been undergoing accessibility interventions. During the mapping exercise, both people with disabilities and rehabilitation professionals discussed the underlying reasons for the contextual barriers and ideas to tackle the root problems. Lack of disability and accessibility awareness was mentioned by both groups. In the words of one participant:

*“...the problem is culture and the fact that people that are in charge, our governors and city mayors...that they know about planning so when a new building is going to be designed … you know, when people visit other countries they come back saying that there a lot of people in wheelchairs...and it is not that...the thing there is that people with disabilities live a normal life, they are not stuck at home and they have accessibility for everything...here we see a person with a disability and we have to tie them to a rope like Tarzan … ” [Female, Person with disability].*

Educating others on disability awareness and accessibility was mentioned as an urgent strategy needed. Lack of disability awareness results in exclusion as explained by a participant with a disability:

*“I think it is better that the parking spots for persons with disability are marked with a cone...even if you have a difficulty to get off the car to move the cone...you can scream, ask someone the favor … , if the cone is not there, people will use it...really, the problem is the citizen’s culture...including thinking that the person with disability is only the wheelchair user” … [Female, Person with disability]*

The rehabilitation professionals had done an experiential exercise with public officials from the municipality and shared:

*“We did an awareness exercise with officers from public infrastructure development...we crossed a light - we had them use wheelchairs - one of them was in the middle of the crossing when the light changed to red..cars honked...this person later called the people in charge to inquire why the duration of the green pedestrian light was so short” [Male rehabilitation professional].*

**Personal-Community-System:** limited mention to current policy as an influential factor were present. Only one mentioned a specific case to use legal appeal (tutela mechanism in Colombia) to drive change: “Some fellow students are going to help me to legally appeal so the university’s accessibility is fixed” [Male, Rehabilitation professional].

**Fig. 3:**
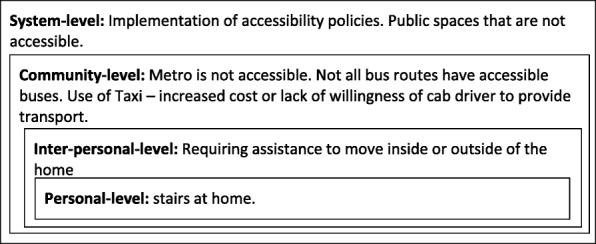
Illustrative example of interaction of factors at multiple levels. In this example a participant describes the challenges he/she faces when trying to get to medical appointments on time [Figure developed by the authors]

The above-mentioned results were jointly presented by researchers and study participants to a group of stakeholders in Envigado in a community forum. We used examples such as the one illustrated in Fig. 3 to facilitate the discussion and identify the barriers operating at the different levels. Coordinated actions between the local government, academia, people with disabilities, and other organizations were discussed as a needed strategy to overcome the hindering factors at the different levels.

## Discussion

People with disabilities and community stakeholders in Envigado, Colombia identified a range of community assets that are not always enjoyable under equal conditions due to barriers at the personal, interpersonal, community, and system level. Some of the most limiting barriers include those at the personal and home level, where people with disabilities typically depend on caregivers, family or neighbors to accomplish routinely tasks such as leaving the home or independently accessing a community asset. Factors at the community level include inaccessible transportation, pathways, and built environment, in addition to negative attitudes towards people with disability in the community. Common strategies to overcome personal and community level barriers to access assets include relying on others for mobility or trying to accomplish tasks mostly on their own, with a significant toll on every day’s life. By using a qualitative approach alongside with participatory mapping, we were able to identify multi-level barriers and facilitators from the perspectives of people with disabilities and community stakeholders, providing evidence that could inform future interventions.

Our findings show that at the personal level, people with disabilities in Envigado typically lack appropriate mobility devices and training on how to use them, limiting not only access to external assets but also enjoyment and use of their home. Access to appropriate assistive technology and associated services (e.g. training on how to use the device) have been acknowledged as requirements to achieve all human rights and to meet the Sustainable Development Goals (SDGs) [35, 36]. Training in assistive technology use is needed to use it effectively in one’s own context, to reduce barriers [37]. The CRPD states that within the right to personal mobility (article 20) the person must receive training in the use of an assistive technology devices [3]. In addition, and specific to wheelchairs, the World Health Organization Guidelines on Appropriate Wheelchair Provision clearly recommends that users - and family members when applicable - receive appropriate training on how to use the device and navigate the environment [38]. The benefits on adequate access and use of technology are supported by a recent study in the US showing that home accessibility modifications positively impact the life of lower-income older adults with functional disabilities by allowing them to continue to live independently at home [39, 40].

At the community level, accessibility across the physical environment (e.g. street and sidewalks pathways, public transportation, public and private buildings and facilities) is a major factor that contributes to exclusion by hindering the ability of people to use community assets. As reported previously, not only there is a general lack of accessibility; but, when actions are implemented towards progress, they are frequently done incorrectly (i.e. very steep ramps, inappropriate use of accessible parking spots) [41]. According to the national disability registry, 46% of people with disabilities encounter barriers on the street that hinder their personal mobility and daily activities [19]. There are also reports of people with disabilities in urban settings in Colombia, spending more financial resources on taxis than their peers without disabilities due to the lack of accessible buses [42]. More broadly, our results also align with evidence from other contexts in Latin America where students with disabilities identified that the infrastructure to get to and at the university and attitudes of professors and administrative staff pose difficulties for the full enjoyment of the university and to exercise their right to education [41, 43–45]. In high income settings, such as the US and Denmark, barriers at the community level prevail where people with disabilities report difficulties in using health care facilities and green spaces, respectively [46, 47]. In Austria, barriers that hinder wheelchair users from using community assets were ground conditions, curbs stones, and gradients [48]. In Sweden, in addition to the previous barriers, difficulties in services/assistance and attitudes/support also negatively impacted participation [37]. An additional consideration is the interaction between spatial-temporal factors, where assets despite being available cannot be accessed or enjoyed unless the adequate support is present at the right time. Worldwide accessibility to the physical and built environment is still a significant issue, that is heightened when interacting with in space and time with personal-level barriers [16].

Our results illustrate how the interaction of personal, interpersonal, and community factors impacts the participation of people with physical impairment. Limited access to community assets increase social isolation and exclusion of people with disabilities [49]. Our results, as the ones by Hammel et al., have implications for assessing contextual facilitators and barriers that affect participation. Our work provides evidence to support system changes, and identifies targets to prioritize, coordinate, implement, and enforce actions at the municipal level in Envigado [13]. As exclusion is multidimensional, there is a need to have tailored, coordinated actions that address the different levels of barriers and aim at ameliorating the potential negative outcomes of their interaction [50]. It is important for service providers, funders, and policymakers to understand that contextual changes at the community level, supported by system’s changes and personalized individual intervention can positively influence participation of people with disability in the community [13].

Neither people with disability nor rehabilitation providers specifically mention the effects of current policies or their lack of enforcement. The legal framework in Colombia is progressive and clearly mandates accessibility as a human right, as stated in the constitutional reform from 1991, in the Disability Law that was enacted after the ratification of the CRPD, and the subsequent National Disability Public Policy Plan [51]. Article 9 of CRPD mandates State Parties to: “take appropriate measures to ensure people with disabilities access, on an equal basis with others, to the physical environment, to transportation, to information and communications, including information and communications technologies and systems, and to other facilities and services open or provided to the public, both in urban and in rural areas” [3]. The experiences captured in this study demonstrate that the implementation of this legal framework is still at its infancy. This is also evident in a critical review of the first Colombian report to the United Nations, where the organization urges the State to guarantee universal accessibility to all and to especially address the needs at the territorial level [52]. In Colombia, especially in rural areas tangible actions to accelerate the implementation and enforcement of accessibility are urgently needed [2, 49]. Even though there are regulatory mandates in place, lack of enforcement leads to limited sanctions and accountability [53]. People with disabilities should be the experts called to lead the implementation, monitoring, and evaluation of the legislation [54].

Capacity building among different stakeholders to understand disability issues and inclusive development is urgently needed [2]. Our findings support that in general the society’s awareness about disability is very low. Social and attitudinal barriers result in intersect with the physical and contextual barriers [1, 3]. In Colombia there is evidence that coordinated and persistent advocacy actions by citizens can foster change. Specifically, a situational analysis of the interaction between public transportation infrastructure, civil society organizations, and the rulings of the Colombian constitutional court in Bogota demonstrated how persistent actions at the individual level, using a system’s level tool, can result in a positive change in accessibility [55]. As an example, in New Zealand to improve accessibility of new construction in accordance with the current legislation, people with disabilities and their organizations have acted as consultants to plan, design, and conduct practical simulation or usability evaluation [14]. Awareness raising is imperative as negative attitudes have been proven to be a barrier to achieving change (accessibility) [53]. For example disability officers at universities in South Africa justified not modifying buildings because of historical heritage value and cost of modifications [53]. A previously proposed strategy to raise awareness is exposing architecture students to experiential exercises to understand the importance of accessibility and universal design was found positive [56]. This type of educational activities should be promoted more since architects are key to advocates for inclusive design and to build an environment that is accessible to all members of society [56]. Importantly, this type of strategies must be implemented with caution. Research has shown that putting yourself in someone else’s shoes might have an opposite effect than intended - it is recommended that more inclusive curricula incorporates contemporary representations of disability, insider expertise, and awareness of strategies for challenging discrimination and promoting disability justice [57]. To be able to have disability-related training in universities - and appropriate access to education for students with disabilities - university professors need to be better trained on universal learning design [41, 43, 44]. The government must have a leading role taking the measures as mandated by Article 8 in the CRPD on awareness-raising: “To combat stereotypes, prejudices and harmful practices relating to people with disabilities, including those based on sex and age, in all areas of life” [3].

As advances in the legal framework in Colombia have been substantial, there is need for further engagement of the community at large. Advocacy efforts should be targeted towards gaining stronger political support and commitment of financial resources to implement and deliver inclusive community assets [2]. In addition to financial support, it is important to shift efforts towards inter-sectoral collaboration and articulation between disability experts. This can lead to system-wide approaches to achieve inclusive results and not only isolated programs that tackle one type of barrier [2]. Recent evidence demonstrates that this is possible: the government of Tajikistan in less 10 years with political will, technical assistance, finance and inter-sectoral effort was able to establish a national rehabilitation system according to the SDGs [58].

### Study limitations and ongoing work

Results of this study should not be used alone to inform policies and programs since it does not represent the views of different types of impairments and experiences of disability. Taking into consideration only one impairment type yields to interventions that do not impact positively all the community [59]. Additional barriers are faced by other type of impairments, for instance how people with hearing impairments have difficulties accessing the web [60], watching television [61], or going to the movies [62]. On the other hand, people with visual impairments may face additional barriers accessing digital education resources [63] and people with cognitive or intellectual impairments may face barriers understanding written information [64]. The focus of this community asset participatory mapping was the assessment of resources in the built environment; therefore, there was no focus on access to information on the digital arena (i.e. digital accessibility). There is also evidence of a growing digital divide between those with and without disabilities and with the aging population [65]. On the other hand, this study only explored experiences in adults, the perspectives of younger, older, or veterans and ex-combatants with disabilities is needed to be able to tackle and overcome barriers in a fully inclusive and universally accessible way [39, 66]. This is the experience of a sub-urban setting in Colombia, which may be similar to other settings in Colombia. However, due to the geographical diversity of the country, there may be differences in the experience of people with disabilities who live in rural contexts, and future engagement with the rural community is needed. The majority of our participants were women. This may be an indication of better rapport with women with disabilities than men since in our context there are more men with physical disabilities [19]. With regards to rehabilitation professionals, there are more women than men serving in health services in the country [67]. Future work may combine the qualitative data gathered by the group to date with Geographical Information Systems and survey data to further explore discrepancies between perceived access to assets vs actual access (e.g. geographical availability of assets) [68].

Results from this project were disseminated to the larger community through a forum where the main findings were presented and discussed with public officials, academics, people with disabilities and advocacy organizations. As a consequence of engaging the community in this project, a community research group was created including community leaders, undergraduate students, and the co-authors of this work to further collect evidence on the current state of the accessibility to public transportation in the municipality. This resulted in a meeting of community leaders with decision-makers within the Metro system and the municipal Secretary of Transportation to discuss barriers evidenced through this work and promote urgent actions to address them. Additional engagement with the local government was done to access the local registry of people with disabilities. This database will be analyzed to identify the location of people with disabilities to inform the deployment of accessible public buses.

## Conclusions

People with disability in Envigado, Colombia face significant barriers mainly related to the right to personal mobility and accessibility to fully enjoy community assets. Engagement of people with disabilities in research alongside with relevant stakeholders provides an opportunity to identify gaps in the implementation of actions as well as strategies that are relevant for the community. Identification of the interactions between barriers that limit access to community assets, allows people with disabilities, organizations, academia, and the local authorities to propose coordinated evidence-informed actions to advance the full participation of people with disabilities.

## Data Availability

The datasets generated and analysed during the current study are available from the corresponding author on a reasonable request.

## Abbreviations

CRPD: Convention on the Rights of Persons with Disabilities
SDGs: Sustainable Development Goals
